# Geometric Conformability of 3D Concrete Printing Mixtures from a Rheological Perspective

**DOI:** 10.3390/ma16216864

**Published:** 2023-10-26

**Authors:** Luiza R. M. de Miranda, Balša Jovanović, Karel Lesage, Geert De Schutter

**Affiliations:** Magnel-Vandepitte Laboratory, Department of Structural Engineering and Building Materials, Ghent University, Technologiepark 60, 9052 Ghent, Belgium; luiza.miranda@ugent.be (L.R.M.d.M.);

**Keywords:** cementitious materials, 3D concrete printing, rheology, buildability

## Abstract

The effectiveness of 3D concrete printing (3DCP) relies on understanding the rheological properties of cementitious materials and their time-dependent evolution. These materials exhibit shear-thinning viscosity, an elastic region, and both static and dynamic yield stress, which are challenging to balance in 3DCP. Layer deformation can be caused by factors such as self-weight, the weight of subsequently deposited layers, and the stress induced by the nozzle pressing. Starting at the level of a single filament, the final geometrical conformity of a 3D-printed object is the sum of individual filament conformities. Hence, the control of layer deformation during the printing process is critical. The failure of 3D-printed objects can occur due to two primary mechanisms: material failure, which occurs when the material’s strength is exceeded, resulting in fracture or uncontrolled deformation; and stability failure, where the object cannot retain equilibrium of forces. These mechanisms often interact; extensive deformations resulting from material failure can lead to stability loss, or conversely, stability loss generates local excessive stresses leading to material failure. The governing mechanism depends on various factors, including material and process characteristics, as well as the transient nature of material properties, print strategy, and object design. With this in mind, this research aimed to broaden the understanding of the connection between rheological material properties—primarily yield stress—and the geometric conformability of printed objects. Experimental tests were conducted on pastes using a rheometer, and correlated mortars, allowing for the evaluation of realistic extrusion properties.

## 1. Introduction

The effectiveness of 3D concrete printing (3DCP) relies on understanding the rheological properties of cementitious materials and their time-dependent evolution. These materials exhibit shear-thinning viscosity, an elastic region, and both static and dynamic yield stress. Achieving the correct equilibrium of these properties is a challenge within the domain of 3DCP [1].

During the hydration process of cementitious materials, physical and chemical changes occur when they come into contact with water, contributing to their structural development. Key mechanisms responsible for structural build-up include hydrate nucleation, formation of C-S-H bridges, and flocculation due to colloidal interactions [2].

Yield stress is a crucial parameter representing the minimum force required for the material to flow like a liquid. It significantly influences the performance and printability of 3D-printed structures. High yield stress or plastic viscosity can lead to nozzle blockages, hindering the extrusion process [1]. On the other hand, dynamic yield stress directly affects flowability, underscoring the importance of maintaining a low dynamic yield stress for high-quality printing.

The yield stress also plays a crucial role in the compressive forces during printing and the resistance encountered during deposition. It represents the material’s load-bearing strength at the time of printing and its ability to withstand deformation under nozzle pressure while maintaining shape and stability [3,4]. Achieving optimal buildability requires the yield stress to increase linearly over time, or the elastic modulus to increase exponentially for specific component geometries [5,6,7]. The recommended yield stress range for stability during printing is 100 Pa to 10 kPa [7].

Layer deformation can be caused by factors such as self-weight, the weight of subsequently deposited layers, and the stress induced by the nozzle pressing. Starting at the level of a single filament, the final geometrical conformity of a 3D-printed object is the sum of individual filament conformities [7]. Hence, the control of layer deformation during the printing process is critical.

Predicting and achieving geometric conformity in 3D printing would be straightforward if the printed material remained unchanged during the process. However, the complexity arises due to structural changes in the material and varying yield stress values in subsequent layers at the same printing time. This results in different levels of deformation for each layer, causing irregular shapes for wall elements that were initially designed as straight walls [3]. To ensure proper printing, the pressing force applied by the nozzle must be carefully controlled. Excessive force can cause increased flow rate and material spreading, leading to inaccuracies in the final object’s dimensions. On the other hand, insufficient force can compromise adhesion between layers, risking the overall structural stability of the printed object.

Throughout the printing process, gravity-induced stress in a given layer increases with the number of layers printed above it. Balancing this stress with the yield stress of the material requires that the material’s properties always remain stronger. This means that the strength of the material from the start must either be greater than the highest gravity-driven stress reached at the end of the printing process, or increase fast enough to dominate gravity [7].

Failure of 3D-printed objects can occur due to two primary mechanisms: material failure, where the material’s strength is exceeded, resulting in fracture or uncontrolled deformation; and stability failure, where the object cannot retain equilibrium of forces. These mechanisms often interact, with extensive deformations due to material failure leading to a loss of stability, or loss of stability causing material failure due to local excessive stresses. The governing mechanism depends on various factors, including material and process characteristics, as well as the transient nature of material properties, print strategy, and object design [8].

With this in mind, this research aimed to broaden the understanding of the connection between rheological material properties—primarily yield stress—and the geometric conformability of printed objects. Experimental tests were conducted on pastes using a rheometer, and correlated mortars, allowing for the evaluation of realistic extrusion properties.

## 2. Materials and Methods

In this study, the evaluation of four print-enabling pastes with varying rheological properties was conducted using both rotational and oscillatory rheometry techniques. Following that, an upscaling process was implemented from the matrix to the mortar scale to enable in-line measurements of rheological properties, in addition to performing small-scale printing trials.

### 2.1. Materials and Mixture Design

To permit the examination of a wide range of behaviors, the formulation of the printable mortars was carried out with the underlying premise that they would possess similar initial yield stress but exhibit distinct yield stress development over time.

As binders, CEM I 52.5 N (by Holcim, Brussels, Belgium) Portland cement (PC) and ground granulated blast furnace slag (GGBS), conforming to EN 15167–2, were used. Calcium sulfoaluminate (CSA) cement (i.tech ALI CEM GREEN^®^ by Italcementi, Bergamo, Italy) was used as an alternative binder, replacing part of the PC amount in the mixture, as well as a setting accelerator. The chemical composition and physical properties of the employed binders are detailed in Table 1. Other chemical admixtures used were a polycarboxylic ether-based superplasticizer (PCE) (Masterglenium 51 by BASF, Ludwigshafen, Germany) in liquid form with a solid content of 34% and a density of 1.10 ± 0.03 g/cm^3^; and a methyl hydroxypropyl cellulose (HPMC) viscosity modifying agent (Tylose^®^ MOT 60000 YP4 by Shin-Etsu, Niigata, Japan), which is water-soluble, non-ionic and in powder form with a density of 1.1–1.5 g/cm^3^.

The structural build-up in cementitious materials is caused by attractive interactions between cement grains leading to flocculation and the precipitation of hydration products at contact points between cement grains. When cement-based materials are at rest, distinguishing reversible processes (flocculation) from irreversible processes (hydration bonding, like C-S-H bridges) is challenging [2]. It is possible, however, to create artificial differences in thixotropic and hydration contributions within the mortar to enforce a particular process to govern the structural build-up phenomenon during a given timeframe. For this reason, nano clay and calcium nitrate tetrahydrate were additionally selected as mix components.

Calcium nitrate is a commonly employed admixture in concrete production, primarily used as an antifreeze agent to lower the freezing temperature. However, this admixture exhibits multifunctional properties at a relatively high temperature of 20 ± 2 °C. Apart from its antifreeze properties, it can accelerate the concrete setting process (dosage of 0.2–1% by mass of binder), neutralize the plasticizer impact while retaining the same rheological features (0.2–1%), retain long-term strength (1–3%), and inhibit corrosion (3–4%). This study employed a dosage of 1% of calcium nitrate tetrahydrate ≥98% (extra pure, density 1.82 g/cm^3^) as an accelerating admixture [9,10].

Attapulgite nano clay is a highly purified magnesium aluminum silicate clay known for its superior properties, including high specific surface area and strong adsorption capacity, which can enhance the characteristics of cementitious materials in their fresh state [11]. The addition of a small amount of nano clay increases thixotropy by creating attractive forces that result in a stable microstructure and higher viscosity at rest. This, in turn, enhances the attractive van der Waals forces, thereby improving thixotropy [12]. In this study, a highly purified thermally treated attapulgite nano clay (by Faber&VanderEnde, Almere, The Netherlands) was used as a thixotropy enhancer. The nano clay particles had an average length of 1.75 μm, an average diameter of 3 nm and a specific surface area of 150 m^2^/g. The addition of nano clay was 0.5% by weight of the total amount of binder.

The aggregates used had a maximum particle size of 2 mm and a specific gravity of 2.65. Their random dense packing fraction was determined experimentally, similar to the procedure described in [13]. A container with 78 mm diameter and 75 mm height was filled with sand and, after the top surface was leveled, it was vibrated for 2 min at 50 Hz, with a 0.5 mm amplitude and a 1.5 kg mass applied above the sample. The material was tested as (i) complete sand, (ii) 125–250 µm fraction, (iii) 315–500 µm fraction, and (iv) 500–1000 µm fraction. The dense packing fraction of the aggregates was found to be 0.62.

When applicable, some features were chosen as fixed parameters, namely (i) PC and GGBS in a proportion of 50:50, (ii) water-to-binder ratio of 0.35, (iii) aggregate-to-binder ratio of 1.5, (iv) HPMC dosage of 0.1% by weight of binder (bwob), and (v) fixed initial spread diameter of 145–155 mm determined by the flow table test conforming to ASTM C-1437. Any necessary adjustment to meet these requirements was made varying the PCE dosage.

The nomenclatures of mortars are related to the main material used to promote structuration, either by enhancing thixotropy or by accelerating hydration. Therefore, SLAG is the reference mixture, CSA has the addition of 10% of calcium sulfoaluminate cement in substitution of PC, NIT has 1% of calcium nitrate tetrahydrate, and NC has 0.5% of nano clay. The final composition of the four designed mortars is detailed in Table 2.

Finally, to ensure reproducibility, the mortars were prepared in a Hobart mixer according to a mixture protocol. First, all dry materials were mixed for 30 s at 140 rpm. Then, the water, pre-mixed with superplasticizer, was added and mixing was continued for 2 min. Finally, HPMC was added to the mixture and mixing was continued at high speed (285 rpm) for another 2 min. In the mixture with nitrate, this material was added dissolved in the mixing water with PCE; and in the mixture with nano clay, this material was added to the dry mixture with extra mixing time for better dry dispersion.

### 2.2. Rheological Measurements

From a thixotropy perspective, it has been demonstrated that assuming no water absorption, predicting the evolution of mortar or concrete only requires an understanding of how the interstitial cement paste changes over time. As a result, if the mechanical effect of the non-colloidal particles (such as sand or coarse aggregates) is to increase the yield stress by a certain factor, then their impact on the structuration rate of the paste will increase by the same factor [5].

Taking this statement into account, it sufficed for the rheological measurements to be performed on cement pastes. The MCR 102 (Anton Paar, Graz, Austria) with a cross-hatched parallel plate geometry of 50 mm diameter was used for all rheological measurements, which were programmed with the aid of the RheoCompass measuring software version 1.2. This geometry was chosen to prevent surface-related artifacts and possible minor sedimentation effects in the measurements, by ensuring that cement samples are sheared in the bulk. The MCR 52 was used with a helix geometry to mix the cement pastes at a controlled shear rate.

A similar mixing protocol to the one described in Section 2.1 was employed to ensure the reproducibility of experimental data. First, all dry materials were deposited into the small cup of the MCR 52 rheometer and quickly homogenized by hand. In the case of the mixture with nano clay, this dry mixing was conducted for a longer time. After the addition of the water pre-mixed with superplasticizer (and nitrate, when applicable), the components of the cement paste were mixed for 60 s at a fixed low shear rate (10 s^−1^). Then, a higher shear rate (300 s^−1^) value was imposed for another 60 s. As all rheological tests were performed in a much lower shear rate range, this value was considered sufficient to ensure good dispersion of the solid particles, minimizing shear degradation effects during the measurements.

Finally, a paste sample was transferred to the MCR 102 rheometer, properly placed in between the cross-hatched parallel plates and left to rest for the necessary time before the beginning of the test with the equipment already in measurement position. The protocols of each test with the corresponding resting times before measurements are described in Section 2.2.1 and Section 2.2.2. A 2 mm gap between the plates was set and a solvent trap geometry cover was used to minimize evaporation during the tests. All experiments were performed at 20 °C and repeated at least three times for a proper representation of the average values.

#### 2.2.1. Flow Curve

The flow curve of the paste was determined by a shearing protocol. Since cement-based materials exhibit thixotropic behavior, it is well known that their rheological response to shear depends on the flow history. Then, it must be ensured that all tests have the same initial dispersed state. Therefore, before starting the measurement, a pre-shear of 100 s^−1^ was imposed for 60 s. After 30 s of rest, the shear rate was logarithmically decreased from 100 to 1 s^−1^ within 40 s, followed by a logarithmic increase from 1 to 100 s^−1^ for another 40 s. The steady-state criterion was selected to provide equilibrium shear stress at a given shear rate and the data were recorded 20 times per second. Plastic viscosity and dynamic yield stress were determined by considering the curve results of each paste that adequately fit the Bingham model Equation (1).

(1) $$\tau =\tau_{0}+\mu \dot{\gamma }$$

where *τ* is the shear stress (Pa), $\tau_{0}$ is the yield stress (Pa), $\dot{\gamma }$ is the shear rate (s^−1^), and μ is the plastic viscosity.

#### 2.2.2. Structural Build-Up

The structural build-up of fresh cement-based materials is commonly assessed using the static yield stress evolution from a flow onset test and the storage modulus evolution from a small amplitude oscillatory shear (SAOS) test. Both techniques provide valuable insights as they arise from different mechanisms. The static yield stress reflects colloidal interactions and interparticle bridging due to hydration products, while the storage modulus indicates the strength of C-S-H and/or ettringite bridges formed between cement particles. Using both methods allows for more precise and comprehensive monitoring of structural build-up [6,10,12].

To achieve a reference state, the fresh pastes were initially sheared at 100 s^−1^ for 120 s and allowed to rest for 30 s to dissipate any residual stresses that may have been caused by mixing. Then, continuous SAOS measurements were carried out for 60 min at an amplitude of 0.0005% and frequency of 1 Hz.

The flow onset test consists of subjecting the material, initially at rest, to a low and constant shear rate until the corresponding shear stress reaches its peak or plateau value required to initiate flow, i.e., the static yield stress (SYS) [5]. The SYS is crucial for the stability of extruded layers in the absence of formwork, as it enables them to support their weight and withstand the vertical pressure from subsequent layers.

As the structure of a material is time-dependent, SYS values can be measured over time, and these values can be plotted against the material’s age to create a characteristic curve that is then used to determine the structural build-up rate parameter (*A_thix_*) [14]. Knowing the structural build-up rate is necessary to ensure that there is no plastic collapse when placing consecutive layers and its behavior may follow either a linear or non-linear trend. In this study, *A_thix_* determination was made by fitting the SYS characteristic curve to Perrot’s exponential thixotropic model [15] Equation (2).

(2) $$\tau_{0}\left(t\right)=A_{thix}t_{c}\left(e^{t/t_{c}}-1\right)+\tau_{0,0}\;$$

This model presents *A_thix_* as a structuration rate, $\tau_{0,0}\;$ is the yield stress of the material with no time at rest, and it has a characteristic time $t_{c}\;$ that marks the beginning of the exponential increase of the yield stress, associated with a non-negligible solid volume fraction linear increase, the value of which is adjusted to obtain the best fit with experimental data.

Due to the highly transient rheological behavior of cementitious materials, it is a challenge to get reproducible results when analyzing the SYS evolution; hence, the need for a measurement protocol. First, the paste sample was pre-sheared at 200 s^−1^ for 120 s to guarantee the same destructed initial state. After the desired resting time, a constant shear rate of 0.05 s^−1^ was applied to the sample. A new sample was mixed for each measurement to prevent inaccurate values of SYS due to multiple disturbances of the material on a single sample. For each investigated age, at least three tests were conducted.

### 2.3. Isothermal Calorimetry Tests

Calorimetric tests were conducted to assess the progression of hydration reactions, providing a more comprehensive understanding of the impact of hydration on the time-dependent rheological properties of concrete. The tests were performed on cement paste using a TAM AIR isothermal heat conduction calorimeter manufactured by TA Instruments, New Castle, DE, USA). For each test, a sample weighing 14 g was used, and the test started 10 min following the initial contact between water and the binder. Heat evolution values were recorded by the equipment at 30 s intervals for 24 h. To ensure the reproducibility of the results, the experiment was repeated twice.

### 2.4. Yield Stress

**Figure 1:**
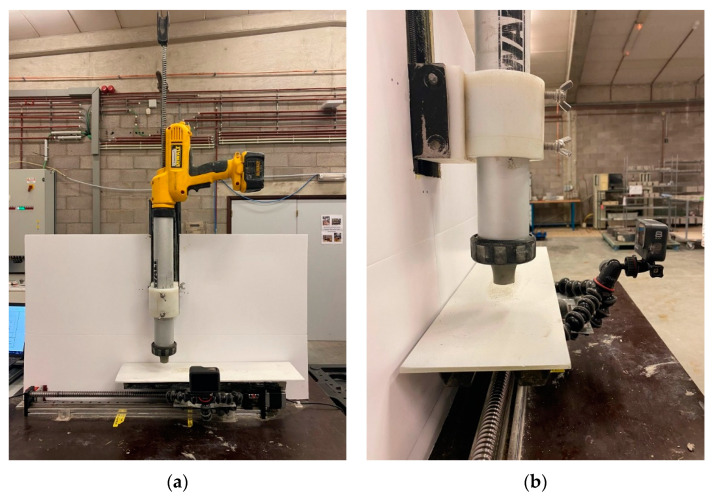
(**a**) Front and (**b**) side view of custom-made setup for small-scale printing, consisting of an electric silicone gun positioned on top of a moving platform. A digital camera was placed on the platform allowing for real-time recording of the printing.

The utilization of some conventional in situ tests may not be trivial due to the fast-structuring nature of 3D printing concrete. To access the yield stress of the studied mortar at the nozzle exit, the slugs test was performed as described in [16]. This test considers the inherent characteristic of 3D-printable mixtures to undergo discontinuous flow when freely flowing from the nozzle due to gravity-induced forces. The authors have shown that the morphology of the slug can be effectively linked to the rheological characteristics of the material as the necking phenomenon represents the initiation of flow. A small container was positioned on top of a scale and below the small-scale printing device (Figure 1) and 10 slugs were collected with the same printing parameters used in the making of the specimens. The yield stress was then calculated using Equation (3):

(3) $$\tau_{c}=\frac{g}{\sqrt{3}S}m_{S}$$

where $\tau_{c}\;$ is the yield stress, *S* is the nozzle section (= $\pi r^{2}$ in this study), *g* is the earth’s gravitational constant, and $m_{S}$ is the slug mass. According to [16], the mass of the slug is primarily determined by the yield stress and, to a lesser extent, viscosity, whereas surface tension is usually negligible compared to the yield stress.

### 2.5. Printing Experiments

For the printing experiments, a small-scale approach was used. The setup (Figure 1) was a custom-made apparatus allowing for the printing of subsequent layers only in the x-direction. The setup encompassed an electric silicone gun, with a maximum extrusion rate of 9.7 mm/min and a maximum normal load of 2900 N, positioned above a moving plate. The maximum translation in the x-direction was 40 cm and in the z-direction it was 15 cm. The gun had a maximum volume of 0.65 l, and up to 10 layers were printed with a 2 cm circular nozzle, a horizontal speed of 15 mm/s, and a vertical speed of 1.5 mm/s. The sample was cut right after printing and the cross-section profile was used to measure the layer deformation. The system allowed for changes in the process parameters such as layer offset, building rate, and nozzle geometry.

### 2.6. Overall Deformation Assessment

One of the primary challenges of 3D concrete printing is ensuring the geometric conformability of the printed elements. Achieving accurate and precise shapes is crucial, necessitating the measurement of layer deformation during the printing process. This deformation can be caused by factors such as self-weight, the weight of subsequently deposited layers, and the stress induced by the nozzle pressing.

To address this challenge, a comprehensive protocol was developed to measure the height deformation over time during the printing process. This protocol involved utilizing a digital camera mounted on the moving platform of the custom-made setup for small-scale printing (see Figure 1b). The camera allowed for the recording of the entire printing process. A sequence of time-lapse pictures was captured at one-second intervals during the deposition of each layer. Subsequently, a Python program was developed to analyze these images.

**Figure 2:**
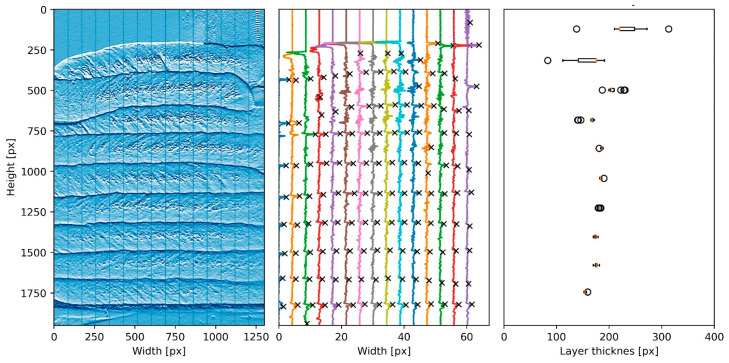
Example of measurement of the layer height deformation throughout the printing process: partition of the image into 50 pixels-wide vertical segments, identification of peaks for delimitation of the layers’ interfaces, and measurement of overall layer height with standard deviation.

The first step in the analysis involved converting the pictures to grayscale and applying a convolution filter to enhance the visibility of the horizontal lines that demarcate the layers’ interfaces. The images were then divided into vertical segments, each consisting of 50 pixels in width. For each segment, the average pixel intensity along its height was determined. By identifying the peaks that indicated the layer boundaries for each vertical segment, an average value was calculated, with a standard deviation (Figure 2). This process effectively demarcated the interface between adjacent layers.

**Figure 3:**
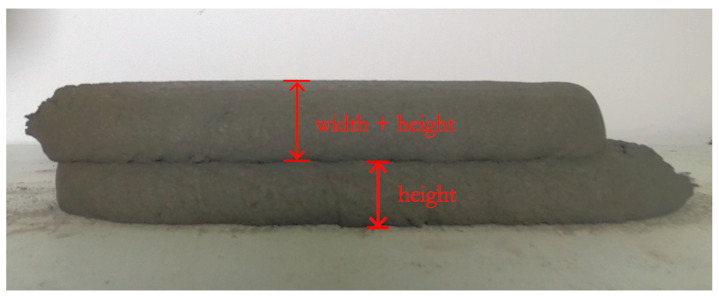
Challenges in identifying the correct layer interface: the positioning of the camera often makes it unclear how to differentiate between height and width in the top layer; thus, the deposition of the subsequent layer becomes necessary for an accurate assessment of the correct value.

With the recorded layer height values from each picture, it became possible to evaluate the progression of deformation over time. However, a boundary condition needed to be established due to the positioning of the camera at the level of the platform. As is depicted in Figure 3, distinguishing between height and width measurements could be non-trivial. Consequently, the first recorded value for the height was only considered once a layer had been deposited on top of the previous one, delimitating the correct interface and setting the boundary condition. Notably, during the post-processing stage, the code only recorded the height variation of the layers corresponding to the current printing stage, thus eliminating any undesired misidentification of values caused by issues such as poor lighting or printing equipment contours.

## 3. Results and Discussion

### 3.1. Flow Curve

The rheological behavior of cement-based mixtures is crucial for their flow properties during 3DCP. In this study, four print-enabling pastes were formulated aiming for different evolutions of rheological properties. The objective was to ensure that the mixtures possessed the same initial yield stress but exhibited varying degrees of yield stress development over time, thereby enabling the examination of a distinct range of behaviors.

**Figure 4:**
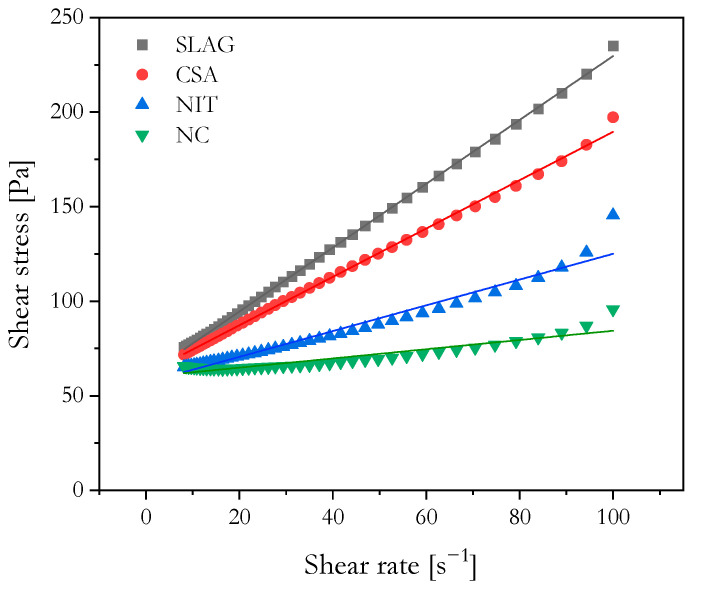
Flow curve of analyzed mortars. The line represents the fit to the Bingham model.

The flow curves for the four pastes are presented in Figure 4. Their dynamic yield stress and plastic viscosity, which were determined by applying Bingham fitting to the downward portion of each flow curve, are shown in Table 3. A similar dynamic yield stress value across all mixtures was observed, which supported the initial premise.

Yield stress is crucial for 3DCP performance, impacting the material’s printability. A high yield stress or plastic viscosity can lead to nozzle blockage, hindering the extrusion process. Maintaining a low dynamic yield stress is essential for high-quality printing. Managing the interplay between different rheological properties is a major challenge in 3DCP, as increased static yield stress and structural build-up rate often accompany increased viscosity, potentially compromising pumpability [1].

While the yield stress values of all the mixtures were similar, the SLAG and CSA mixtures exhibited significantly higher plastic viscosities. Several factors could have contributed to the higher viscosity observed in the CSA mixture. CSA cement promotes greater surface interactions compared to PC, leading to the faster formation of denser hydrates like ettringite. This increased formation enhances friction between adjacent particles when subjected to shear force, thereby elevating the plastic viscosity [17].

On the other hand, the NC mixture enhanced thixotropy, increasing static yield stress and the structuration rate while minimally affecting viscosity. This behavior has been proven to improve the printing performance of cement-based composites [11]. The effectiveness of NC as a nanomaterial depends heavily on the dosage and dispersion method employed. Although the flow curve results may not have directly revealed the effect of NC, the structural build-up, accessed through SAOS and flow onset tests (discussed further in Section 2.2.2), was higher for the NC mixture when compared to the SLAG and NIT mixtures.

It should be noted that attapulgite NC can increase viscosity but also exhibits high shear thinning capacity, where viscosity substantially decreases under sustained or higher shear rates. The influence of NC on viscosity at a given strain rate may be less significant than its influence on static yield stress. Its thixotropic effect becomes apparent when the material is at rest, leading to an increase in yield stress due to the formation of a network resembling a “house of cards”. However, under steady-state flow conditions, this network progressively breaks down as clay particles align with charged surfaces, resulting in a decrease in apparent viscosity [18].

Studies have shown [19] that the addition of NIT in pastes can reduce viscosity at lower concentrations (up to 2%), while higher concentrations tend to increase viscosity, likely due to increased paste temperature. The effectiveness of NIT also depends on the type of cement used, with higher efficiency observed in cement containing more belite.

### 3.2. Structural Build-Up

**Figure 5:**
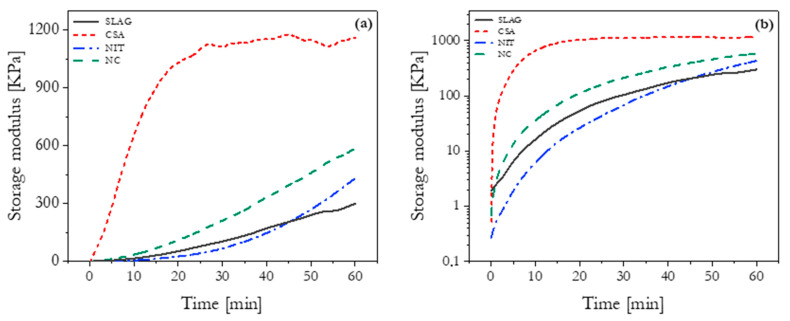
Evolution of storage modulus with time under a strain amplitude of 5 × 10^−4^% in (**a**) linear and (**b**) logarithmic scales.

Figure 5 presents the storage modulus data, with the right figure showing the evolution over time and the left figure using a logarithmic scale for better visualization of the early-stage behavior. In the first 40 min, the NIT mixture had the slowest storage modulus evolution, followed by SLAG, NC, and CSA, which had the highest value. After 40 min, NIT surpassed SLAG and approached NC’s value. It is important to note that the CSA mixture showed a sharp increase in storage modulus within the first 10 min, flowed by a plateau, indicating quick consumption of the added calcium sulfoaluminate cement and ettringite production [20]. In contrast, the reaction of the other mixtures occurred at a slower rate, making it not possible to observe the plateau for the remaining three mixtures within the test duration, supporting CSA’s high stiffening characteristics.

While the storage modulus curves for SLAG and NC seemed to be approaching a plateau, the NIT mixture continued to exhibit slightly linear growth. This suggested a slower hydration rate for NIT but a tendency to reach high values of storage modulus, indicating a potential for stronger structural formation. Isothermal calorimetry data (see Section 2.3) supported this observation, as a shift in the slope of the cumulative heat curve occurred around 1.5 h, with a sharp increase surpassing the other mixtures at approximately 4.5 h after water addition.

The initial storage modulus values of CSA and NC were similar, indicating their structuring characteristics. However, the CSA mortar showed a sharp increase within the first few minutes, while NC followed a slower trend. The rapid increase in storage modulus for CSA, along with subsequent plateauing at a high level, indicated the quick formation of a percolated network. The rapid structural build-up rate could be attributed to the presence of ye’elimite in the CSA cement, which undergoes immediate reaction without a period of slow reaction [21].

In the case of the NC mixture, it showed a higher initial storage modulus compared to SLAG and NIT, indicating a more rigid structural formation, likely due to inter-particle interaction forces induced by the finer particles of NC. The smaller particle size of NC may enhance attractive colloidal interactions, leading to faster development of storage modulus during the very early stages of hydration. The continuous increase in storage modulus during the induction period is associated with the formation of C-S-H bridges between cement particles and the dissolution of ions, contributing to rapid structure development [18].

Some studies [18] have reported conflicting results regarding the effect of nano clay on storage modulus evolution. This study found that the NC mixture outperformed NIT and SLAG in terms of storage modulus evolution. However, when considering the flow onset test, the NC mixture displayed a static yield stress value over time that was higher than SLAG but lower than NIT. The complex interplay between colloidal and hydration effects on structural build-up requires further investigation to better understand the discrepancies observed in storage modulus evolution results.

The static yield stress is crucial for assessing the structural build-up of 3D-printed mixtures, as it reflects their ability to maintain shape and stability. When shear is applied, water–cement interaction leads to a gradual increase in shear stress due to the formation of a flocculated structure that resists deformation. The material behaves like a solid until the flocculated structure breaks, and it transitions into a liquid-like state when the shear stress surpasses a critical threshold (the static yield stress) [5].

The flow onset measurement is sensitive to both soft and rigid interactions, unlike calorimetry and storage modulus measurements that only capture rigid interactions governing hydration kinetics [11]. Rigid interactions involve surface-based precipitation and growth of C-S-H, a key hydrate phase in cement. For 3D printing, the cement paste needs a low dynamic yield stress during extrusion and a high static yield stress afterward to retain its shape under subsequent layer loading, distinguishing it from traditional forming methods [22].

**Figure 6:**
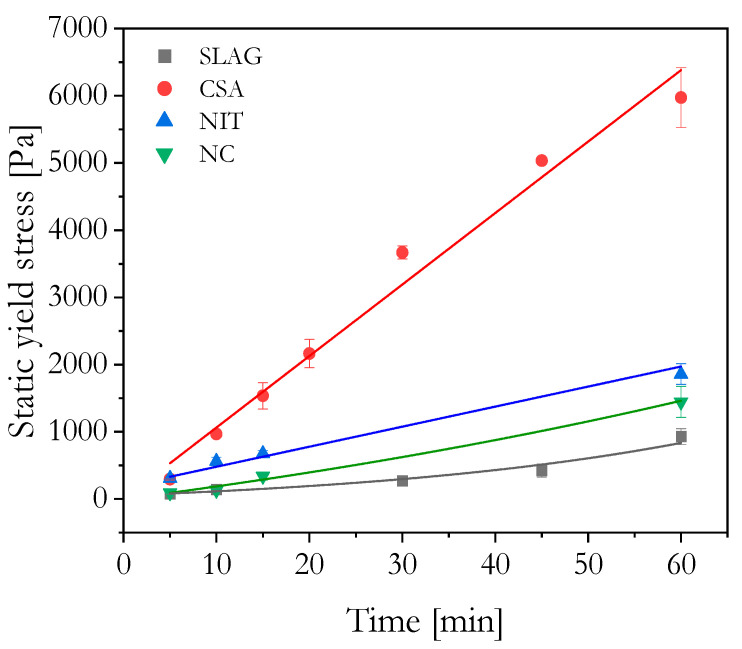
Static yield stress evolution with time at rest. Each measurement was made out of a fresh sample and each point consists of the average of at least three measures. The line represents the Perrot fit.

The influence of CSA on the stiffening effect was demonstrated by the flow onset results. CSA slightly increased the initial yield stress while significantly promoting structural build-up over time (see Figure 6). The increase in yield stress was correlated to the fast early-age hydration of the mixture.

Nano clay improved static yield stress and accelerated structural build-up compared to the SLAG mixture. Mechanisms like particle packing effects, water or polycarboxylate adsorption, and electrostatic attraction contribute to increasing the microstructural flocculation when nano clays are used [18]. Previous studies [23] have demonstrated that systems containing clays result in more resilient flocs that are less susceptible to dynamic disturbances. The influence of nano clays on the structural build-up is mainly attributed to colloidal effects that occur shortly after the flow stops, in contrast to hydration effects which occur over time.

The model proposed by Perrot et al. [15] was used to fit the data of static yield stress evolution as can be seen in Table 4. Among the tested mortars, SLAG exhibited the slowest structural build-up rate, while CSA demonstrated the highest. Slower solidification can be advantageous when precision adjustments and the positioning of printed layers are required. However, it may prolong the overall printing time. On the other hand, rapid solidification and early strength development prove favorable for time-efficient construction, although it can result in nozzle blockage and tearing. Striking the right balance between workability and time efficiency is essential in 3DCP to ensure successful and efficient printing processes.

### 3.3. Isothermal Calorimetry

Isothermal calorimetry is a widely used method to directly measure heat production during cement hydration. The heat generated is linked to the amount of hydration products formed and, consequently, to the strength of the cement. The production of hydration products enhances the yield stress by increasing surface area and promoting C-S-H bridge formation. Monitoring the onset of the acceleration period in the calorimetry curve is crucial for understanding the setting process in 3D-printed materials and can provide valuable processing information [7].

**Figure 7:**
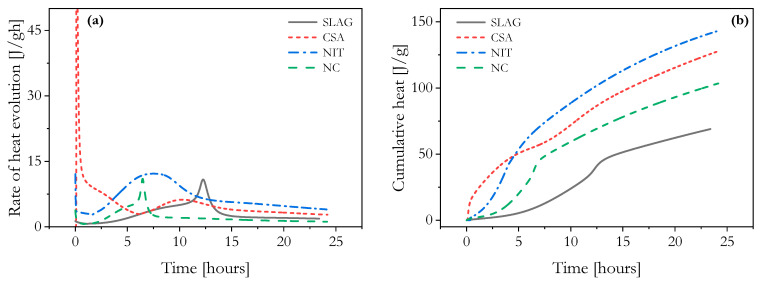
Isothermal calorimetry curves. (**a**) Rate of heat evolution and (**b**) cumulative heat.

The comparison of hydration heat flow curves (see Figure 7) provided valuable insights into the behavior of the NC and SLAG mixtures. Although their shapes were similar, there was a noticeable shift in the position of the peaks, suggesting that the presence of nanoparticles accelerates the hydration of cement. This is mainly because they act as nucleation sites, promoting the formation of C-S-H and increasing its precipitation. As a result, the hydration rate of C_3_S tricalcium silicate is accelerated, and the induction period is shortened [18]. This observation was supported by the results of the flow onset test, where the NC mixture consistently exhibited higher static yield stress values across all tested ages. Additionally, the NC mixture demonstrated a higher structuration under oscillatory shear test compared to the SLAG mixture, further indicating its enhanced structuring capabilities.

In terms of the NIT mixture, the beginning of the flow onset measurements revealed relatively close static yield stress values when compared to the CSA mixture. However, after approximately 10 min, a clear distinction emerged as the CSA curve experienced a sharp increase due to its high structural kinetics.

Portland cement and CSA cement differ significantly in how they undergo hydration, especially regarding the speed at which it occurs. CSA cement experiences a remarkably fast hydration rate, with most of the heat generation happening within the first 2 to 12 h of hydration. The key products formed during the hydration process of CSA cement include crystalline ettringite, monosulfoaluminate, and amorphous alumina hydroxide. The hydration of CSA cement involves three main steps: (i) dissolution of crystalline anhydrous phases, (ii) formation of new phases such as crystalline ettringite and amorphous gels like alumina hydroxide hydrate, and (iii) consumption of free water [20,23,24].

The NIT curve showed the slowest structuration process in the SAOS measurements. However, around the 45 min mark, the NIT curve surpassed the SLAG curve, indicating a higher level of structuration at that stage. Interestingly, these results did not align completely with the calorimetric analysis.

The utilization of accelerators in concrete is known to decrease the setting time and/or accelerate early strength development, particularly at normal temperatures. Dorn et al. [25] found that the addition of Ca(NO_3_)_2_ promotes the formation of ettringite and a nitrate-containing AFm phase, as well as accelerating alite hydration, particularly at their maximum investigated dosage of 5 wt.%. Regarding the silicate reaction of Portland cement, it has been established that Ca(NO_3_)_2_ reduces the time until the primary hydration of alite commences and enhances the rate of alite hydration, and potentially belite as well. Nonetheless, the authors advise against using dosages exceeding 3% for cement pastes and concrete, as they significantly increase the viscosity of mixtures immediately after mixing.

Analyzing the hydration heat evolution, it appeared that both the CSA and NIT mixtures showed higher levels of structuration. This was evident from their initial peak intensity, indicating rapid hydration reactions at a very early age. The CSA mixture, in particular, exhibited a higher intensity peak, supported by the cumulative heat measure, with the CSA curve showing the highest dissolution rate within the first 3 h. The reduced induction period resulted in faster solidification of the mortar, thereby enhancing buildability.

After this initial surge, the slope of the curve diminished, indicating a stabilization period, only to experience another increase at around 10 h. Research has indicated that the type and content of sulfate significantly influence the hydration of CSA systems [17]. One important finding was that a high sulfate content can retard the dissolution of belite and ferrite phases. Consequently, a high sulfate content can accelerate the rate of hydration during the early stages while retarding hydration at later ages. 

The NIT cumulative heat curve, on the other hand, displayed a change in slope direction, with a sharp increase starting around 2.5 h after the beginning of the test, surpassing the CSA curve after 4 h. This aligned with the end of the dormant period and the beginning of the acceleration period. However, it remains unclear why the cumulative heat of the NIT mixture was significantly higher than that of the other mixtures, including CSA. This discrepancy suggested that the reaction process in the NIT mixture underwent a higher degree of acceleration, which did not agree with other experimental findings.

### 3.4. In-Line Yield Stress

The yield stress of the material plays a crucial role in both the compressive forces exerted by the printing and the resistance encountered during the deposition process. It denotes the load-bearing strength of the material at the time of printing, as well as its ability to withstand deformation under the nozzle pressure, maintaining its shape and stability [3,4].

If the material being printed did not undergo structural changes during the printing process, its deformation would simply follow a linear relationship with the resistance it encounters. This would make predicting the final dimensions straightforward and enable more straightforward adjustments to the printing parameters for achieving the desired geometric conformity. However, the challenge here lies not only in the material’s inherent structural properties but also in the variability of yield stress values among successive layers during the printing process.

The slugs test is a way to understand how 3D-printable mixtures behave when they flow out of the nozzle. Gravity plays a role in causing the material to flow unevenly, resulting in a slug-like shape that can be connected to the material’s rheological properties, especially when the flow begins. The slug’s mass is mainly influenced by the material’s yield stress and, to a lesser extent, its viscosity [17]. As aforementioned, the printable mortars in this study were designed under the assumption of having the same initial yield stress but different yield stress development over time, allowing for the examination of a distinct range of behaviors. The values tabulated in Table 5 and the rheological analysis above confirm this premise. The CSA mixture had a somewhat higher value, which was still in the same order of magnitude and stemmed from its high structural build-up rate. The values were in the range of 3D-printable mixtures’ yield stress, previously reported to be 600–2000 Pa [26].

### 3.5. Geometric Conformability of the Layers

#### 3.5.1. Height Variations

In 3DCP, three distinct approaches have been reported. The infinite brick extrusion uses high-yield-stress and low-thixotropy materials to create rectangular layers, simplifying layer geometry control but limiting design freedom to conform to the nozzle’s dimensions. The free-flow deposition strategy relies on low-yield-stress materials and the final geometry is influenced by the interplay between gravity and yield stress, offering limited control over object shape. The layer pressing strategy employs an often circular nozzle and fluid material, providing greater design freedom and compensating for deformation. It allows precise control and self-correction, with the layer width adjustable by varying flow rate, nozzle velocity, or height. However, careful parameter adjustment is essential for optimal results [3,27,28,29].

As previously stated, to accurately measure the height deformation of the printed layers during the 3DCP process, a specific boundary condition was established. The first recorded height value for each layer was considered only after the next layer had been deposited on top of it. This approach ensured accurate measurements by eliminating potential inconsistencies in the height values caused by inaccuracies inherent to the setup and explained above (see Section 2.6). In other terms, the height of the first layer was recorded only after the deposition of the second layer. Consequently, in the case of samples with 10 layers, only nine height values are presented, while samples with 9 layers exhibit eight presented values.

When employing an 8 mm offset, the different mix designs exhibited varying behaviors. The SLAG mixture displayed a significant height variation, with the top layer measuring 6.42 mm and the bottom layer measuring 8.77 mm, resulting in a difference of 27%. This deformation indicated that the pressing force exerted by the nozzle on the layer was higher than the insufficient internal structure strength to resist deformation.

**Figure 8:**
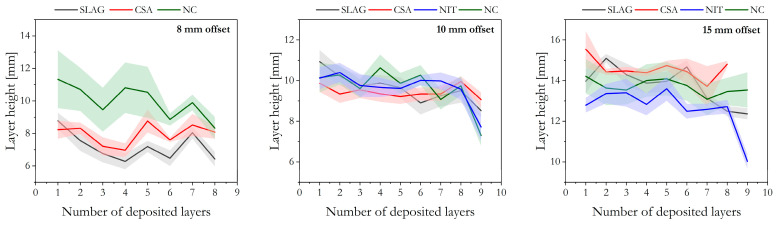
Variation in height of the bottom layer upon deposition of subsequent layers on small-scale printing experiments. The data are presented divided by layer offsets, with the corresponding error band.

Similarly, the NC mixture also exhibited a 27% difference between its top and bottom layers, with the top layer measuring 11.3 mm. As depicted in Figure 8, NC consistently exhibited larger layer sizes compared to the other mixtures, indicating a higher flow rate. Consequently, a greater amount of material was extruded from the nozzle, resulting in the observed larger layer height. Among all the mixtures tested, only the NC mixture exceeded the intended 8 mm offset in the average layer height.

In contrast, the CSA mixture, which has been extensively proven to have high structural build-up properties, exhibited a more stable average height of 7.9 mm for all layers. The difference between the top and bottom layers was only 3.5%, and the final height of the printed element was 63.7 mm, very close to the expected height of 64 mm. Huang et al. [16] found that incorporating CSA in mixtures had minimal impact on initial penetration resistance but significantly enhanced its growth as time progressed. This demonstrated that CSA moderately affected extrudability and provided a notable stiffening effect on PC mortar mixtures, enhancing also buildability.

Moving on to the 10 mm offset, similar analyses can be drawn. Despite the reduced layer pressing compared to the 8 mm offset, the nozzle still exerted some pressing force on the layers. The CSA mixture showed consistent layer sizes with a small difference (1%) between the top and bottom layers. The NIT and NC mixtures had average layer heights closer to the target but exhibited larger differences between the top and bottom layers. Hence, while the layer pressing strategy successfully addressed and controlled the final height difference, it resulted in a lack of control over the single-layer height.

Implementing the layer pressing strategy in 3D concrete printing proves to be an effective solution to rectify potential errors in the vertical position of the printed structure. This self-corrective approach compensates for deformations or creeping of deposited sublayers. The material’s low initial yield stress enables greater flexibility during extrusion, enhancing printing conformability. Additionally, the flow rate can be adjusted to compensate for height variation, ensuring that the final height of the printed object remains within a reasonable margin of the projected value [3].

In the case of the 15 mm offset, all mixtures exhibited smaller deformations due to less layer pressing, as the targeted height was closer to the nozzle diameter. The CSA, SLAG, and NC mixtures demonstrated greater uniformity, while the NIT mixture exhibited the largest deformations and the greatest deviation from the desired final height of the printed element. The differences between the top and bottom layers, as well as the deviations from the target height, were 11% and 8% for SLAG, 2.5% and 4% for CSA, 10% and 5% for NC, and 15.5% and 17.5% for NIT, respectively.

**Figure 9:**
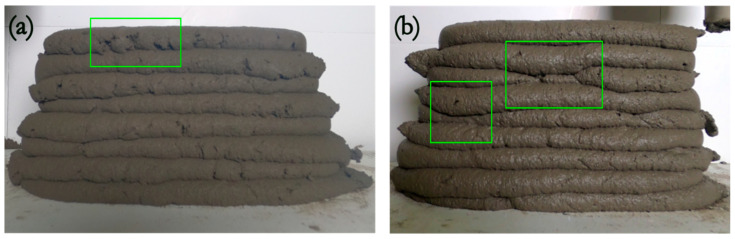
Examples of problems in the printing that can hinder the layer height assessment. The green boxes represent (**a**) surface irregularities and (**b**) flow out of a layer due to eccentricity issues of the small-scale setup.

It is worth emphasizing that the custom-made small-scale printing setup featured a mortar gun that moved upwards via a screw-rotor-type mechanism, which occasionally resulted in slight deviations in the axis of layer placement The absence of a suitable substrate to support the layer caused the material to flow out of the bottom layer. This flow disruption can be considered as a variation in layer height. Since the captured images were taken laterally, the boundary line between layers became distorted and less straight (Figure 9b). Additionally, the detection of layer boundaries can be further complicated by imperfections or cracks in the layers’ surfaces, caused by rapid stiffening or high printing speed. These surface irregularities appeared as peaks, which might have contributed to the larger error margins (Figure 9a).

#### 3.5.2. Width Variation

Although the layer pressing strategy provides benefits, it introduces additional complexity in parameter selection and affects layer geometry. The strategy imposes temporary stress on sub-layers when they are pressed between the nozzle and previous layers. This stress can lead to significant deformation, variations in layer width, potential cracks, and ultimately to structural failure [3]. This failure can occur through elastic buckling, which is a stability mechanism, or plastic collapse, which is a strength mechanism [4].

Suiker et al. [4] in their study revealed that the contact conditions between the wall bottom and the support structure are typically close to fully sticking, which restricts displacements in all directions. As the vertical wall length increases, the normal stress in the wall’s height direction rapidly diminishes due to stress-free boundary conditions and limited wall thickness. The first layer experiences a kinematic constraint from sticking contact, resulting in limited plastic deformations and reduced effective shear stress. In contrast, the second layer from the bottom reaches the yield strength, defining the critical moment of collapse. By testing a small square wall layout, Suiker et al. [4] found that the second layer showed the largest plastic deformations in the wall’s width direction, while the first layer experienced limited plastic broadening due to its kinematic constraint.

Some distinct tendencies were observed when evaluating the three selected scenarios of this study. If the ratio between the layer offset and the nozzle diameter was close to 1, the nozzle applied minimal pressure to the layer during deposition. Consequently, little to no deformation was observed, and the width of the layer at the moment of deposition matched the final size after the completion of the printing element.

However, as this ratio diminished, the nozzle began to apply pressure on the layer generating additional stresses. This pressure exceeded the yield stress of the material at that specific time, resulting in a width broadening, but with reminiscent control of overall structure height.

The layer width then became larger as the material continued to deform due to the combination of the weight of the subsequently deposited layers, nozzle pressure, and gravity-induced stresses. Small layer heights can subject the first sublayer to pressing forces up to 100 times the material weight, inducing significant but localized deformations that jeopardize structural integrity [3]. It is worth noting, however, that there is a maximum limit to this deformation, dictated by the structuring characteristics of the printable mixture.

**Figure 10:**
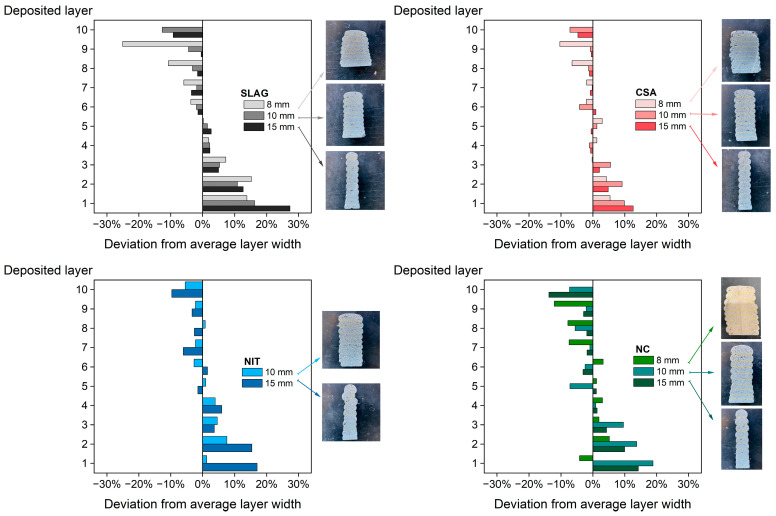
Final layer width of studied mortars. The measures were made from the cross-section of the samples printed in the small-scale printer. The values show the deviation of the measured layer width from the sample’s average width. Positive percentages represent a layer larger than the average, whereas negative percentages represent a layer smaller than the average.

The final layer width of each layer was measured with the aid of a digital caliper in the cross-section of the samples, cut in the fresh state. The resulting layer variation and the final cross-section shape are depicted in Figure 10.

This study focused on investigating the behavior of small-scale samples that were not prone to failure. However, an interesting observation was made when the printing was carried out with an 8 mm layer offset; all the mixtures exhibited a larger width for the second layer compared to the bottom layer. As mentioned earlier, the smaller the ratio between layer offset and nozzle diameter, the greater the pressure exerted by the nozzle on the layer. This indicates that if a large-scale print was conducted, the pressing force would lead to the collapse of the second layer.

It is worth noting that, despite the mixtures having similar initial yield stress and all the printing parameters being kept constant in the experiments, there were relative differences in the average layer width among them (see Table 6), confirming their distinct time evolutions based on their rheological parameters. For the 8 mm offset, the samples had an average layer width of 55 mm, while the 10 mm offset resulted in 45 mm, and the 15 mm offset yielded 35 mm. These findings support the fact that the nozzle offset can indeed be used to tailor the final dimensions of the printed layer.

Upon examining the final layer width, it became evident that the SLAG mixture exhibited the highest variation from the average values, indicating variability in layer width throughout the element height, and it had a significantly larger bottom layer width. This occurred because, when there is pressing, the layer height is mainly determined by the position of the printing nozzle, while various printing parameters, such as robot velocity, material flow rate, and nozzle deposition height, influence the layer’s width.

On the other hand, the NC mixture had the smallest layer width, pointing to the enhanced thixotropic effect of the nano clay, which improved structural stability. The CSA mixture had the smallest standard error of the mean, suggesting a uniform overall layer width.

For the 15 mm offset, where the ratio between layer offset and nozzle diameter was closer to 1, the average final width of all mixtures approached the nozzle diameter size. In this case, the NC mixture had the smallest final width, but notably the largest final width was observed in the NIT mixture, while the SLAG mixture still presented the largest standard error of the mean and thus the greatest difference between the dimensions of the top and bottom layer.

In terms of shape retention, the NC mixture exhibited better behavior as it consistently presented the smallest average of the final layer width across all tested offsets. The CSA mixture demonstrated superior geometric conformability, characterized by a more constant layer width throughout the sample.

#### 3.5.3. Relation between Layer Deformation and Structural Build-Up

During the printing experiments, each layer took approximately 10 s to be printed, with a 15 s interval between successive layers. Consequently, the total printing time for the objects was around 4 min. The slugs test, performed at the nozzle level, resulted in the initial yield stress values presented in Table 5, and the structural build-up rates were determined based on the values in Table 4.

The CSA material demonstrated rapid structuring characteristics, ensuring excellent load-bearing capacity and resulting in objects with consistent layer sizes and minimal deformation along their entire length. The final dimensions were achieved with remarkable precision. However, despite these strengths, the surface appearance of the printed layers could leave much to be desired. Some layers exhibited several tearing defects, which worsened as the printing process progressed. This was likely due to extrusion issues caused by the high stiffness of the rapidly structuring CSA material.

The severity of this problem varied based on the offset used during printing. In the case of an 8 mm offset, the tearing defects were more pronounced, while in the case of a 15 mm offset, they were nonexistent. This difference suggests that the pressing force exerted by the nozzle contributes to surface cracking.

Moreover, the specific geometry of the filament used in 3D printing not only impacts the aesthetics of the final printed object but also plays a crucial role in its structural integrity and long-term durability. The filament’s shape influences bond strength and its ability to prevent the ingress of harmful substances, affecting the overall performance and lifespan of the printed object. The size of the object itself also comes into play. For large objects with longer layer interval times, the print material has a greater opportunity for strength development as each layer solidifies before the next is added. This enables a more substantial build-up of the element, reducing the risk of collapse during the printing process [7,8].

As the CSA material becomes stiffer and no changes are made to the printing parameters during the process, the flow rate decreases as the object is built up. Consequently, the nozzle velocity becomes much higher than the flow velocity, exceeding the nominal speed and leading to tearing. The occurrence of tearing is more likely with an increment in material stiffness or a reduction in the material’s critical strain [8].

The SLAG mixture exhibited the most intriguing behavior among all the studied mixtures. Despite being expected to perform poorly in various aspects, it surprisingly presented the best aesthetic appearance. The layers were uniform, and no significant cracks or tearing were observed during the building process, indicating overall stability. However, it is essential for readers to understand a crucial caveat: this positive observation holds only if one chooses to ignore the initial design specifications. As mentioned earlier, the SLAG mixture deviated the most from the intended geometric features.

Regarding layer height, the layer pressing strategy effectively addressed and controlled the final height difference, ensuring the desired overall height of the printed object. However, this strategy led to a lack of control over the single-layer height, resulting in a deviation of approximately 15% from the target for the SLAG mixture.

In contrast, the NIT and CSA samples demonstrated better uniformity in layer dimensions. Even for the 15 mm offset, the SLAG mixture still exhibited the largest standard error of the mean, indicating the greatest difference between the dimensions of the top and bottom layers. This underscored the importance of considering both material and process parameters when designing 3DCP structures.

## 4. Conclusions

In this study, four distinct mixtures were examined, displaying a range of rheological behaviors: a reference mixture (SLAG), one designed for enhanced thixotropy (NC), another with accelerated hydration (NIT), and a final one comprising both thixotropy and fast hydration features (CSA). Additionally, as the success of 3DCP depends on both material and process parameters, changes in the printing offset were introduced to examine the effect of nozzle pressure on the dynamics of the printing process.

The combination of rapid hydration and thixotropic behavior yielded consistent layer sizes with minimal deformation along their length, resulting in superior geometric conformability, even under the load of the subsequently deposited layers and the influence of the nozzle pressure. However, it was clear that a fast structuration has an upper limit as high stiffness led to extrusion issues and tearing defects in certain scenarios.

Artificial differences in thixotropic and hydration contributions within the mortar were created to enforce a particular process to govern the structural build-up phenomenon during a desired timeframe, and the use of a thixotropic enhancer was more effective in conforming the geometric features.

The change in nozzle offset, and thereby the variation in the pressure exerted on the deposited layers, was found to be a key variable in tailoring the final dimensions of the printed layer. The average final layer widths exhibited an inverse relationship with nozzle pressure.

The reference mixture of this study presented the best aesthetic appearance, but it deviated the most from the intended geometric features. Height variations can compromise overall structural stability, while width variations may require additional work to rectify wall alignment, along with increased material consumption in the case of wider-than-projected layers. These findings emphasize the significance of selecting appropriate material mixtures for specific 3DCP applications to achieve the desired results in terms of structural integrity, aesthetics, and printability.

## Figures and Tables

**Table 1:** Chemical composition and physical properties of binders.

Chemical Composition (wt.%)	CaO	SiO_2_	Al_2_O_3_	Fe_2_O_3_	MgO	Na_2_O	K_2_O	SO_3_
PC	63.48	19.61	5.96	4.13	0.92	0.49	0.64	2.72
CSA cement	41.5	8.70	23.65	1.25	3.69	0.86	-	18.86
GGBS	37.97	35.60	12.13	0.37	7.42	0.74	-	7.24
Nano clay	3.00	50.2	9.8	3.32	9.1	0.6	0.6	-
Physical properties		Blaine [m^2^/kg]		Specific gravity [-]				
PC		408		3.15				
CSA cement		500		3.16				
GGBS		428		2.80				

**Table 2:** Mixture proportions.

Material	SLAG		CSA		NIT		NC	
	Quantity (kg/m^3^)	Volume (%)	Quantity (kg/m^3^)	Volume (%)	Quantity (kg/m^3^)	Volume (%)	Quantity (kg/m^3^)	Volume (%)
PC	397.9	13	726.7	23	394.4	13	396.8	13
Slag	397.9	14			394.4	14	396.8	14
CSA			80.7	3				
Nitrate					7.89	1		
Nanoclay							3.97	0.4
Sand	1193.7	45	1211.2	46	1183.3	45	1190.4	45
Water	278.5	28	282.6	28	276.1	28	277.8	28
PCE	2.39	0.3	4.20	0.3	4.50	0.3	4.84	0.4
HPMC	0.80	0.1	0.81	0.1				
Total weight (kg)	2271.2		2306.3		2260.7		2270.5	

**Table 3:** Dynamic yield stress and plastic viscosity. The rheological parameters were obtained by fitting the flow curve data to the Bingham model.

	SLAG	CSA	NIT	NC
$\tau_{0}$ (Pa)	61	62	57	60
μ (Pa∙s)	1.7	1.3	0.9	0.7

**Table 4:** Parameters relative to the structural build-up obtained by fitting the static yield stress evolution data to Perrot’s exponential model.

	SLAG	CSA	NIT	NC
$t_{c}\;$(min)	33	12	8	44
$\tau_{0}$ (Pa)	64	240	151	69
$A_{thix}\;$(Pa/min)	5	106	32	11

**Table 5:** Initial yield stress value obtained at printing exit through slugs test.

	SLAG	CSA	NIT	NC
$\tau_{0}$ (Pa)	780	860	800	790

**Table 6:** Values of the final layer width of the bottom and top layers, as well as the average width of the printed samples, for the three different layer offsets.

	SLAG			CSA			NIT			NC		
Width (mm)	8 mm	10 mm	15 mm	8 mm	10 mm	15 mm	8 mm	10 mm	15 mm	8 mm	10 mm	15 mm
Bottom	65.92	57.10	42.85	59.16	46.85	38.91	-	46.86	40.20	53.40	49.54	37.91
Top	43.41	42.91	30.55	50.34	39.59	32.93	-	43.72	31.03	47.86	38.63	28.61
Average	57.47	49.72	34.78	55.68	43.11	34.96	-	46.55	35.05	52.16	42.41	33.42
SEM *	2.29	1.20	1.03	0.90	0.71	0.49	-	0.55	0.90	1.14	1.05	0.77

* Standard error of the mean.

## Data Availability

Not applicable.
